# The Effect of Addition Potassium Permanganate on Bond Strength of Hot-Dip Galvanized Plain Bars with Cement Paste

**DOI:** 10.3390/ma16072556

**Published:** 2023-03-23

**Authors:** Petr Pokorný, Vítězslav Vacek, Nikola Prodanovic, Adam Zabloudil, Karel Hurtig

**Affiliations:** 1 Department of Building Materials, Klokner Institute, Czech Technical University in Prague, 166 08 Prague, Czech Republic; 2 Department of Experimental Methods, Klokner Institute, Czech Technical University in Prague, 166 08 Prague, Czech Republic

**Keywords:** corrosion, concrete, hot-dip galvanized steel, permanganate, bond strength test

## Abstract

In this paper, the effect of gradually increasing amounts of KMnO_4_ (10^−4^, 10^−3^, 10^−2^ mol·L^−1^) in cement paste on the bond strength of a plain hot-dip galvanized steel bar was evaluated. The open-circuit potential of HDG samples in cement paste with various additions of MnO_4_^−^ was monitored in order to follow a transfer of zinc from activity to passivity. Furthermore, the influence of the addition of these anions on the physicochemical properties of normal-strength concrete or cement paste was evaluated by means of hydration heat measurements, X-ray diffraction analysis, and compressive strength. The effective concentration of MnO_4_^−^ anions prevents the corrosion of the coating with hydrogen evolution and ensures that the bond strength is not reduced by their action, which was determined to be 10^−3^ mol·L^−1^. Lower additions of MnO_4_^−^ anions (10^−4^ mol·L^−1^) are ineffective in this respect. On the other hand, higher additions of MnO_4_^−^ anions (10^−2^ mol·L^−1^), although they ensure the corrosion of the coating in fresh concrete without hydrogen evolution, but affect the hydration process of the cement paste that was demonstrated by slight water separation.

## 1. Introduction

The use of coatings in the corrosion protection of concrete reinforcement can be an economically acceptable and sufficiently effective option for the construction industry to prolong the service life of structures, especially against the action of de-icing salts [1,2,3]. It is the use of de-icing salts (NaCl, CaCl_2_) in winter road maintenance that causes localized corrosion damage to the conventional carbon steel reinforcement of concrete after overcoming the concrete cover layer [4,5].

From the beginning of the last century to the present day, the possibility of using some potentially effective and, at the same time, economical coatings that are technologically easy to apply to conventional rib reinforcement in concrete has been intensively investigated [6,7]. From this point of view, the possibility of using organic coatings (epoxy coatings, PVC, bitumens, etc.) and coatings produced by the hot-dip galvanizing (HDG) process has been intensively investigated [6,7,8,9]. Despite some shortcomings of epoxy coatings (proven reduced bond strength with concrete, insufficient abrasion resistance, the tendency to crack during long-term deposition below freezing point), their use in corrosion protection of concrete reinforcement is used on a large scale in the USA and Japan (optimal coating thickness recommended 300 µm) [10,11,12,13]. The eventual commercial use of hot-dip galvanized coatings for the same purpose has not reached the same scale as in the case of epoxy coatings. The reason for this is the still contradictory conclusions evaluating the effectiveness of this form of corrosion protection also with regard to the sustainability of the coating process and, of course, the effect on the load-bearing capacity of buildings [14].

In the case of exposure of HDG steel in simulated concrete pore solutions, an initial very intense corrosion attack occurs (anodic corrosion process of zinc oxidation is given as Equation (1)) under the evolution of hydrogen (cathodic corrosion process, see—Equation (2)) [6,8,9,14].

(1) $$\mathrm{Zn}\to {\mathrm{Zn}}^{2+}+2e^{-}$$

(2) $$2H_{2}O+2e^{-}\to H_{2}+2{\mathrm{OH}}^{-}$$

Insoluble corrosion products are formed on the surface of the samples with the majority of Ca[Zn(OH)_3_]_2_·2H_2_O (ZnO/Zn(OH)_2_ phases are also present) [6,8,9,14,15,16]. It is usually stated that mainly this phase (Ca[Zn(OH)_3_]_2_·2H_2_O—calcium hydroxyzincate—CHZ) conditions the transition of HDG steel active corrosion to the corrosion in the passive state (in the case of pH of model pore solutions up to 13.3 ± 0.1) [6,8,9,14,17,18,19]. The formation of Ca[Zn(OH)_3_]_2_·2H_2_O in model concrete pore solutions is described by the following Equations (3)–(5) [18,19,20]:

(3) $$2\mathrm{Zn}{\left(\mathrm{OH}\right)}_{2}+2H_{2}O+\mathrm{Ca}{\left(\mathrm{OH}\right)}_{2}\to \mathrm{Ca}{\left[\mathrm{Zn}{\left(\mathrm{OH}\right)}_{3}\right]}_{2}\cdot 2H_{2}O$$

(4) $$2\;{\left[\mathrm{Zn}{\left(\mathrm{OH}\right)}_{4}\right]}^{2-}+{\mathrm{Ca}}^{2+}+2H_{2}O\to \mathrm{Ca}{\left[\mathrm{Zn}{\left(\mathrm{OH}\right)}_{3}\right]}_{2}\cdot 2H_{2}O+2{\mathrm{OH}}^{-}$$

(5) $$2\mathrm{Zn}+\mathrm{Ca}{\left(\mathrm{OH}\right)}_{2}+6H_{2}O\to \;\mathrm{Ca}{\left[\mathrm{Zn}{\left(\mathrm{OH}\right)}_{3}\right]}_{2}\cdot 2H_{2}O+2H_{2}$$

However, the results of some studies suggest that ZnO/Zn(OH)_2_, which fills the pores between the plate-like CHZ crystals, plays an important role in the protective properties of the corrosion products of zinc and HDG steel in simulated concrete pore solutions [14,21].

The most significant problem discussed in connection with the use of hot-dip galvanized concrete reinforcement is the initial corrosion under hydrogen evolution, which increases the porosity of the cement paste at the phase interface. This fact may be reflected by a reduction in the bond strength of the HDG reinforcement with concrete [20,22,23].

If the initial cathodic corrosion reaction under hydrogen evolution (Equation (2)) in fresh concrete is prevented, then the potential of hot-dip galvanized coatings as corrosion protection of concrete reinforcement could be exploited, regardless of the limitations of the bond strength of such coated reinforcement with concrete. In this matter, further surface treatment of hot-dip galvanized concrete reinforcement is discussed [24,25,26,27], or corrosion inhibitors can be added into fresh concrete in order to prevent corrosion of the hot-dip galvanized coating under hydrogen evolution.

The results of some papers discuss the effect of water-soluble chromates (CrO_4_^2−^) in cement (100–200 ppm) on the inhibition of coating corrosion under hydrogen evolution [28,29,30]. Although Cr^VI^ compounds are toxic [31,32] and, therefore, unacceptable for the construction industry, it has been verified that the addition of CrO_4_^2−^ (CrO_3_) to the simulated concrete pore solution effectively inhibits the corrosion of the hot-dip galvanized coating under hydrogen evolution [33]. Nitrite (NO_2_^−^) [34], diethanolamine (C4H11NO2) [35], or molybdenates (MoO_4_^2−^) [36] may be effective alternatives in this regard.

Recently, the effect of KMnO_4_ addition on the reduction in corrosion of hot-dip galvanized steel in the environment of simulated concrete pore solutions was verified (the effective concentration of MnO_4_^−^ in the model concrete pore solution was 10^−3^ mol·L^−1^) [37]. While this concentration was verified to ensure the corrosion of the hot-dip galvanized coating without hydrogen evolution, it was also verified to slow down the actual anodic process (RDHE—rapid dissolution and hydrogen evolution).

However, the applicability of this inhibitor in construction practice is conditional on testing its effect on the bond strength of hot-dip galvanized reinforcement with concrete, which has not yet been professionally assessed. It is necessary to verify whether the corrosion of the hot-dip galvanized coating in real concrete is actually inhibited by the development of hydrogen and, therefore, whether the bond strength is reduced as a result (the emerging hydrogen increases the porosity of the cement paste at the phase interface [6,8,9,14,20,21,22]). From this perspective, it is also necessary to verify the influence of the composition and precipitation of new phases (Mn-rich) of corrosion products on the bond strength of HDG steel with concrete [20].

Although it is assumed that KMnO_4_ does not affect the hydration of normal-strength concrete [38], the results of the measurement of the heat of hydration of concrete in the case of the addition of graded amounts of MnO_4_^−^ ions, as well as the changes in its compressive strength (cubic specimens) are discussed in the remainder of this paper. Changes in the composition of the crystalline phase of the hydrated concretes, again with graded addition of MnO_4_^−^ ions applied to the mixtures, are also evaluated by means of X-ray diffraction.

It is clear from the literature that it has not yet been verified in detail whether the addition of KMnO_4_ affects the hydration of conventional silicate cement. Through SEM image analysis and X-ray diffraction analysis of the composition of cement slurry samples, it was found that KMnO_4_ additions do not negatively affect the morphology of the hydrated sample and also that the cement slurry contains conventional phases such as ettringite, portlandite, and other usual minerals [38]. It can be expected that MnO_4_^−^ in the alkaline environment of fresh cement mix will rapidly convert to MnO_4_^2−^ and then to MnO_2_. The presence of the very stable MnO_2_ phase is unlikely to affect the hydration of cement (i.e., to be involved in the hydration reactions of the clinker minerals). Moreover, the significant oxidation potential of this substance will not affect the hydration of cement but may inhibit the action of any organic-based plasticizing additives used.

The toxicity of KMnO_4_ [39,40] and its possible leachability from concrete are not evaluated in this paper.

## 2. Materials and Methods

For the individual tests applied in this test program, specimens were produced from cement paste and normal strength concrete (NSC). In both cases, Portland cement CEM I 42.5 R (Českomoravský cement, a.s., Mokrá-Horákov, Czech Republic) was used. The oxide representation of the Portland cement used is shown in Table 1. Cement paste was used in the specimen production for cohesion tests and X-ray diffraction analysis, while NSC was used in the determination of the heat of hydration and verification of the changes in cubic compressive strength.

The cement paste specimens were prepared according to the mix given in Table 2 (w/c: 0.35); the normal strength concrete (NSC) specimens were prepared according to the recipe given in Table 3 (w/c: 0.55). The consistency of the cement paste was set to ensure that the material completely fills the space between the plain bars and reference electrodes (stainless steel). A w/c ratio of 0.42 was used for this purpose. More water would mean its subsequent separation from the cement paste. Since the use of a plasticizer was not considered, the consistency of the concrete had to be adjusted using only water. Unlike cement paste, concrete contains filler (fine and coarse fractions of aggregate), which adsorbs part of the mixing water on its surface. At the same time, such a consistency had to be achieved so that the concrete could be properly compacted. A w/c of 0.55 was used for this purpose.

Graduated amounts of KMnO_4_ were added to each mixture (cement paste, NSC) according to Table 4. An ordinally graded amount of MnO_4_^−^ anions was added and dissolved in distilled water (8.5 µS·cm^−1^). The addition of this treated premixed water was continuous during the actual mixing of the mixture.

The batch (cement paste and NSC) was mixed by a standard device (concrete mixer GIFOS MB 80, speed rotation 30 rounds per minute—rpm) for 10 min to achieve homogeneity. The mixture cast in steel cubic molds with a plain bar positioned and fixed in the center axis was compacted in two phases. Firstly, the mold was half-filled with concrete, then compacted for 5 min, and then the second half was filled and compacted again for 5 min. The specimens were vibrated by a hand device (Eibenstock EBR 125.1, 10,000 rpm -Elektrowerkzeuge GmbH Eibenstock, Eibentsock, Germany). The standard devices were intentionally used to achieve conditions expected in practical applications.

All test bodies (cement paste, NSC) were aged for one day in a humid atmosphere (65% RH; 20.5 ± 1 °C) and then stored under water (distilled water).

**Figure 1:**
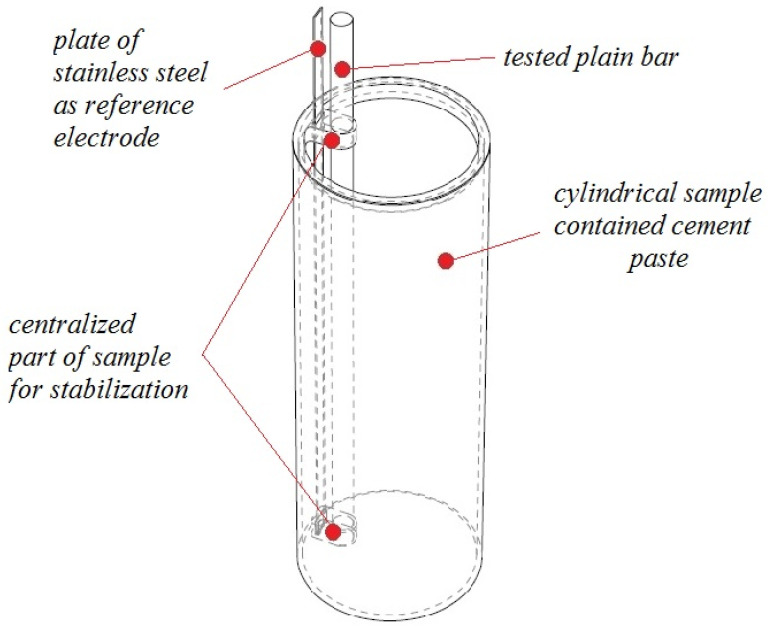
Modeling of samples designed for measurements during E_corr_/FeCr18Ni9 and bond strength of hot-dip galvanized plain bars in cement paste.

**Figure 2:**
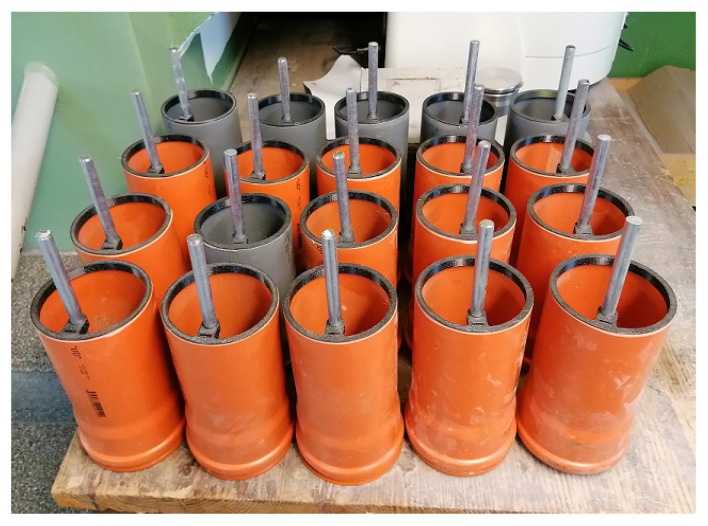
Detailed view of test fixtures for bond strength tests before filling with cement paste.

For the modified bond strength test, a pull-out test method was used (loading and evaluation of test results according to RILEM RC6 [39] and ASTM C234-91a [40]), cylindrical specimens of PLA Filament (see Figure 1 and Figure 2) with wall thickness 0.5 cm, inner diameter 9.5 cm, and height 21.0 cm were fabricated. The anchorage of the plain bars to the axis of the test cylinders for bond strength tests was provided on opposite sides of the cylindrical mold (holes for the plain bars to be threaded through). At the same time, an additional narrower hole was created (top) in the same areas to guide a plate of stainless steel (FeCr18Ni9-Goodfellow metals, Huntingdon, UK) as a reference electrode for E_corr_ measurements. The distance between the surface of the plain bar and the plate of stainless steel (reference electrode) was 5 mm. The bottom of the mold was closed with a cap (PE) which had a hole for the plain bar to be threaded through. These cylindrical bodies and the adjacent caps (bottom side of the mold) were printed on a 3D printer (Prusa i3MK3S+ - Prusa Research, Prague, Czech Republic). In this way, a total of 28 bodies were created (7 for each intention—see Table 4). The test specimens were placed above the water surface in an open grid (closed plastic box—95% RH). The open-circuit potential of FeCr18Ni9 plates versus SCE (saturated calomel electrode) was measured in simulated concrete pore solutions at pH 12.8 with graded amounts of MnO_4_^−^. The readout of the potential of the stainless steel (multimeter—OWON B35 (T)) plate versus SCE was taken after approximately 30 min of exposure (see Table 5). For the actual expression of the E_corr_ time course, a conversion to the SCE reference value was performed using the average measured value (last detail in Table 5). With gradually increasing amounts of MnO_4_^−^ in simulated concrete pore solutions, values of potential difference between (FeCr18Ni9)/SCE increase. An increase in a similar range of values (as shown in Table 5) was also verified in the cement paste used to make the cylindrical samples.

OWON B35 (T) was used to measure E_corr_ HDG samples in cement paste cylindrical bodies using stainless steel (FeCr18Ni9) as reference electrode (all E_corr_ measurements). The resulting solids were designed not only for E_corr_ time evolution measurements but also subsequently for (after 28 days of concrete aging) modified plain bars with cement paste bond strength test.

**Figure 3:**
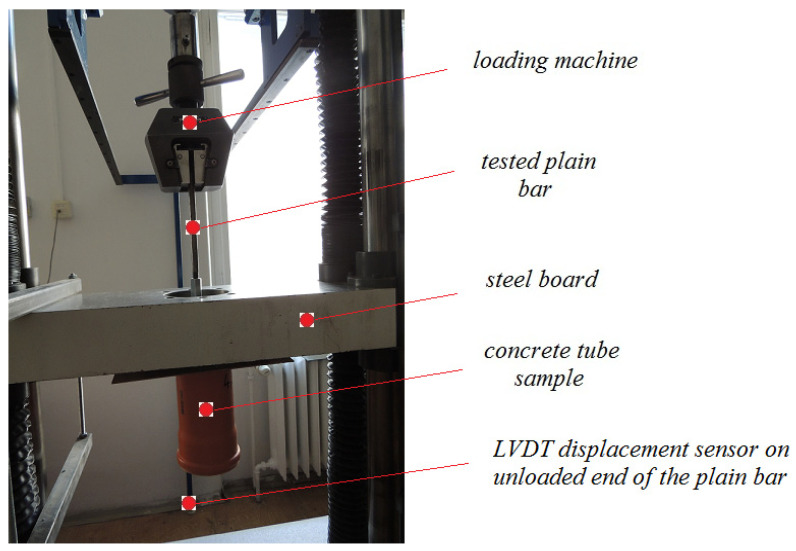
Experimental setup of pull-out test of bond strength between plain bar and cement paste.

The bond strength between the plain bars and a cement paste is determined by measuring the bond stress slip (LVDT sensor—Micro-Epsilon Messtechnik GmbH, Ortenburg, Germany) on the unloaded end of plain bars by loading device TIRA test 100 kN (viz Figure 3). The test was controlled by displacement of the unloaded end of a reinforcing bar. A constant loading rate of 0.005 mm s^−1^ was applied. The setup of the pull-out test is displayed in Figure 3. The test was terminated after reaching a slip of 1.6 mm.

The chemical composition of steel is verified by GD-OES (HORIBA Yobin Ivon—HORIBA Jobin Yvon IBH, Ltd., Glasgow, UK) since both silicon and phosphorus affect the formation of an HDG layer (viz Table 6).

The coating of the hot-dip galvanized steel surface of the plain bars was then imaged by optical microscopy—OM (cross-section). Preparation of the actual samples for OM analysis was based on grinding (LaboPol—2 automatic grinder, Struers GmbH, Willich, Germany) and subsequent polishing (diamond polishing paste). For grinding, abrasive papers of roughness P60—P2400 were used.

The hydration process was monitored by a modified test—instead of conventional hydration heat, the temperature was measured (semi-adiabatic conditions). The composed of thermocouple was enclosed in the middle of a cubic mold (a_c_ = 300 mm) and fixed inside a larger cube. The space between the two was insulated (a_i_ = 100 mm) by a polystyrene layer (isolated system). The temperature of the inner system, composed of curing and eventually hardening normal strength concrete, was measured in 5 min for 11 days. In total, four samples of NSC were measured (Table 4). The average air temperature in the room where the cubes were stored was t = 20.5 ± 1 °C.

The phase composition of the mix of cement pastes (different amounts of MnO_4_^−^, see Table 4) is evaluated using X-ray powder diffraction (PAN analytical X’Pert3 Powder using CuKα radiation over the angular range 5–90°) and a semiquantitative analysis is performed using RIR (reference intensity ratio) values.

Subsequently, the cubic compressive strength (ac = 150 mm) was measured on NSC samples for each mixture (reference sample and MnO_4_^−^ graded samples according to Table 4). A total of seven samples were generated for each intent. The measurements were performed after 28 days of concrete maturation and were carried out on a TIRA test machine 100 kN.

## 3. Results and Discussion

The discussion of the results of the experimental program presented in this paper is included in the following Sections: Section 3.1, Structure of HDG coating; Section 3.2, E_corr_ measuring; Section 3.3, Modified bond strength test; Section 3.4, Measuring of hydration heat of concrete samples; Section 3.5, X-ray diffraction analysis of concrete specimens; Section 3.6, Compressive strength test of concrete cubic samples.

### 3.1. Structure of HDG Coating

**Figure 4:**
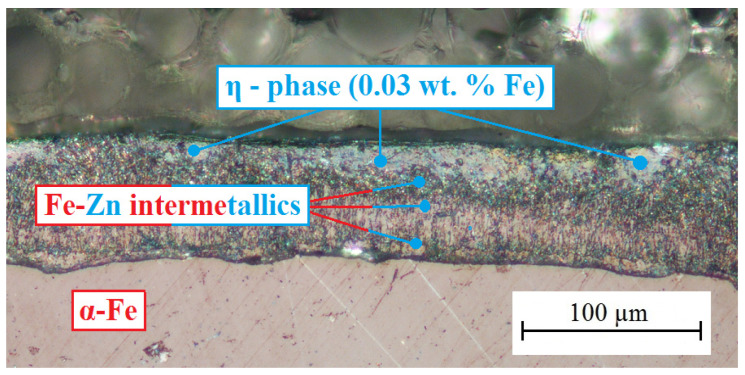
Cross section of hot-dip galvanized coating on the surface of plain bars.

Figure 4 shows a cross-section of the hot-dip galvanized coating on the surface of the plain bars. These specimens were used for the E_corr_ measurement and bond strength tests.

With respect to Figure 4, it is clear that the average coating thickness is about 60 µm. The formed hot-dip galvanized coating is uniform, and no defects (sintering, dust, lumps, etc.) were detected [41]. The composition of the steel, in particular the content of Si, P, and C, has a major influence on the structure of the hot-dip galvanized coating on the steel. For this reason, a GD-OES analysis of the steel composition was carried out (see Table 6). Based on the results of the steel composition, it can be concluded that the Si content lies in the Sebisty region (0.15 wt.%–0.25 wt.%), which conditions the formation of the coating with all intermetallic phases (inner layer of the coating—see Figure 4) [20,41,42]. Thus, a thin layer of the phases Γ (Γ and Γ1), the richest in the presence of iron and formed closest to the steel surface, is present [43,44]. Above this layer, a much thicker layer of δ phases (δ_1k_ and δ_1p_) appears [45,46], followed by a palisaded very thick bore of the ζ phase (FeZn_13_) [20,41,42,47,48]. From the results of the cross-section imaging of the surface of the hot-dip galvanized coating, it is clear that the outer layer is formed by the η phase layer (solid solution of iron in zinc—iron content up to 0.03 wt.%). In most cases, the thickness of this layer is more than 10 µm, which, according to the literature sources [6,8,17,24], is sufficient for the transition of corrosion of HDG samples to the passive state (formation of a sufficiently thick Ca[Zn(OH)_3_]_2_·2H_2_O layer, verified in simulated concrete pore solutions). The carbon (0.11 wt.%) and phosphorus (0.01 wt.%) content in the steel do not affect the structure of the excluded coating [49].

It is clear that the outer layer of the HDG coating is composed of the η phase, with only a local presence of the ζ phase (FeZn13 intermetallic). It can be expected that when such coated steel is embedded in concrete, the surface of the specimen will be sparing (see above for information), but in areas with an exposed outer layer of the ζ phase, there will be an increase in the total volume of hydrogen evolution (see Equation (2))—a lower hydrogen overvoltage on the surface of this phase compared to pure zinc (or η phase) [6,8,17,42,50,51].

### 3.2. E_corr_ Measuring

**Figure 5:**
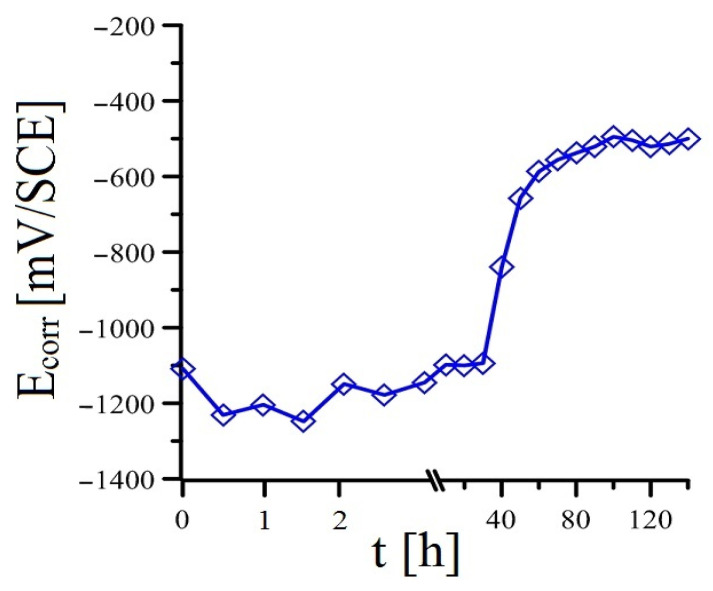
Evolution of open-circuit potential (E_corr_) in samples of HDG steel in concrete without addition of MnO_4_^−^—(mean values).

**Figure 6:**
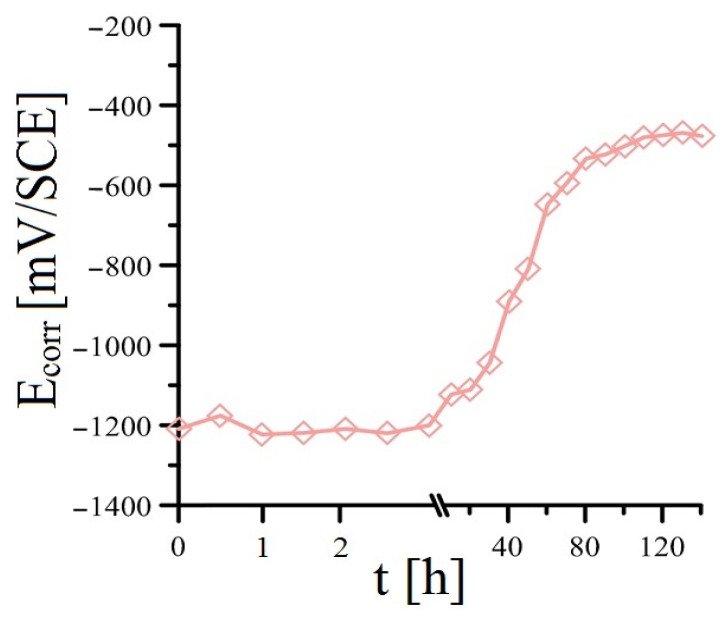
Evolution of open-circuit potential (E_corr_) in samples of HDG steel in concrete with addition of MnO_4_^−^ (Mn^(1)^)—(mean values).

**Figure 7:**
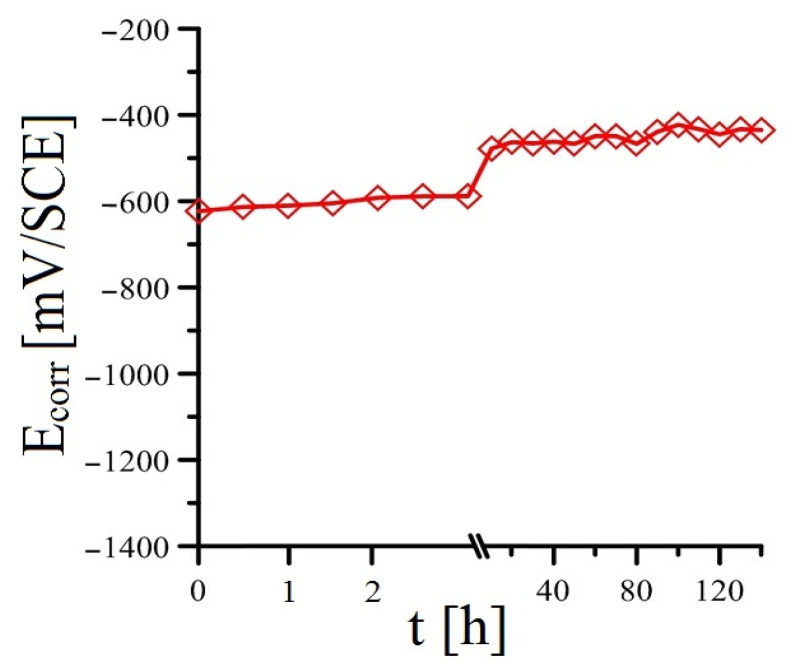
Evolution of open-circuit potential (E_corr_) in samples of HDG steel in concrete with addition of MnO_4_^−^ (Mn^(2)^)—(mean values).

**Figure 8:**
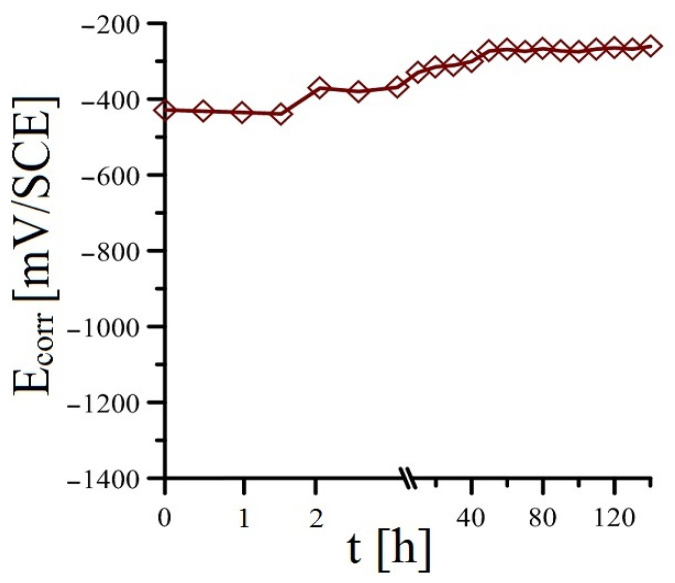
Evolution of open-circuit potential (E_corr_) in samples of HDG steel in concrete with addition of MnO_4_^−^ (Mn^(3)^)—(mean values).

Before the actual modified bond strength test of plain bars with cement paste (cylindrical bodies), the time dependence of E_corr_ was measured, where a stainless steel plate (FeCr18Ni9) was chosen as the reference electrode. The measurements were carried out for 144 h, and the results for each intent are shown in Figure 5, Figure 6, Figure 7 and Figure 8. The measured potential was converted to SCE. It is evident that the measured E_corr_ HDG values of the samples in the individual mortars correlate reasonably well with the measured E_corr_/ACLE HDG values of the samples in model concrete pore solutions with identically graded MnO_4_^−^ anion content [52]. The time dependence of the spontaneous corrosion potential of hot-dip galvanized steel samples in cement paste without the addition of MnO_4_^−^ anions (Figure 5) and with the addition of only 10^−4^ mol·L^−1^ (Mn^(1)^ substitution—see Figure 6.) is very subordinate. It is clear that the lowest addition of KMnO_4_ used in this experimental program does not prevent the corrosion of the coating under hydrogen evolution (hydrogen evolution ceases only after about 40 h of exposure) [14,21,42,53,54]. Hydrogen gas is evolved when the E_corr_ is below—990 mV/SCE (pH = 12.8, t = 25 °C), which is the calculated E_eq_ (equilibrium potential) according to Equations (6) and (7) [42].

(6) $$E_{\mathrm{eq}}=E^{0}-\frac{\mathrm{RT}}{\mathrm{zF}}\cdot \ln\left(\frac{a_{H_{2}\cdot a_{{\mathrm{OH}}^{-}}^{2}}}{a_{H_{2}O}^{2}}\right);{\;E}^{0}=-0.828\;V$$

(7) $$E_{\mathrm{eq}}=-0.828+0.059\;\left(14-\mathrm{pH}\right)=-0.059\cdot \mathrm{pH}\;$$

The development of hydrogen gas by the cathodic corrosion reaction (see Equation (2)) increases the porosity of the cement paste at the phase interface, which can result in a decrease in the bond strength of the hot-dip galvanized steel with the concrete [8,9,20,22,42]. The actual corrosion process of HDG specimens in cement paste with the lowest MnO_4_^−^ addition is similar to the reference exposure, according to E_corr_ time dependence measurements. Based on this fact, it can be assumed that the composition of the corrosion products will also be usual with a majority of Ca[Zn(OH)_3_]_2_·2H_2_O (CHZ—see Equations (3)–(5)) [52]. The presence of a coarse layer of CHZ corrosion products can also adversely affect the bond strength of HDG plain steel with cement paste [20]. Although, according to other results, the presence of these corrosion products can increase the cohesion of hot-dip galvanized reinforcement with concrete [55,56]. It is evident that after approximately 40 h of exposure, there is a rapid increase in E_corr_, which can be interpreted by the transition of the hot-dip galvanized coating to a passive state by the formation of a sufficiently dense layer of corrosion products. It can be considered that after approximately 80 h of exposure, the samples corrode at a very low corrosion rate, with cathodic corrosion occurring under the reduction in oxygen to OH^−^ anions—according to Equation (8). Thus, the corrosion rate of the coating is limitingly controlled by the access of atmospheric oxygen to the surface of the samples [57,58]. It is evident that in the case of admixture with the addition of KMnO_4_, the reduction in Mn^VII^ to manganese oxo-compounds occurs at a lower oxidation level [52].

(8) $$O_{2}+2H_{2}O+4e^{-}\to 4{\mathrm{OH}}^{-}\;(E\;\sim +157\;\mathrm{mV}/\mathrm{SCE})$$

Adding a more significant amount of KMnO_4_ to the cement paste (10^−3^ mol·L^−1^ see Figure 7 and 10^−2^ mol·L^−1^ see Figure 8) results in a more significant change (compared to the reference exposure) in the time course of the open-circuit potential. For both exposures of cement paste, the hot-dip galvanized steel corrodes from the beginning of the exposure without hydrogen evolution (according to Equation (2)). For both exposures, the sample of hot-dip galvanized steel in cement paste occupies a potential significantly higher than (-) 990 mV/SCE at the beginning of the exposure. In the case of Mn^(2)^ exposure (addition of 10^−3^ mol·L^−1^ KMnO_4_), the potential increase is rather stepwise, whereas, for Mn^(3)^ exposure (addition of 10^−2^ mol·L^−1^ KMnO_4_), the potential increase is slightly more gradual. It is very likely that on the surface of the hot-dip galvanized steel, there is a reduction (cathodic corrosion process) of Mn^VII^ to oxo-compounds containing manganese in a lower oxidation state from the beginning of the exposure. Based on results reported recently [37,52], it is clear that the reduction takes place in the Mn^II^/Mn^III^ oxidation state. Within the sequence of possible reduction reactions, Equations (9)–(13) can be given [52,59,60,61,62,63,64], and it can be expected that the first step involves the reduction in MnO_4_^−^ anions to MnO_2_ (Equation (9)) [59,60], and then the reduction proceeds to Mn_3_O_4_ (Equations (10) and (11)) [61]. Depending on the conditions, however, the reduction in MnO_2_ can proceed to MnO(OH) (Equation (12)) [52,62], or manganese hydroxides can form on the surface of hot-dip galvanized steel in the Mn^II^ and Mn^III^ moieties (see Equation (13)) [52,63,64]. The surface of the hot-dip galvanized steel sample is then covered with a layer of probably amorphous corrosion products of oxo-hydroxo Mn^II^/Mn^III^ compounds [37,52].

(9) $${\mathrm{MnO}}_{4}^{-}+2H_{2}O+3e^{-}\to {\mathrm{MnO}}_{2}+4{\mathrm{OH}}^{-}(E\;\sim +309\;\mathrm{mV}/\mathrm{SCE})$$

(10) $$2{\mathrm{MnO}}_{2}+H_{2}O+2e^{-}\to {\mathrm{Mn}}_{2}O_{3}+2{\mathrm{OH}}^{-}(E\;\sim +17\;\mathrm{mV}/\mathrm{SCE})$$

(11) $$3{\;\mathrm{Mn}}_{2}O_{3}+H_{2}O+2e^{-}\to 2{\mathrm{Mn}}_{3}O_{4}+2{\mathrm{OH}}^{-}(E\;\sim -308\;\mathrm{mV}/\mathrm{SCE})$$

(12) $${\mathrm{MnO}}_{2}+H_{2}O+e^{-}\to \mathrm{MnO}\left(\mathrm{OH}\right)+{\mathrm{OH}}^{-}(E\;\sim -51\;\mathrm{mV}/\mathrm{SCE})$$

(13) $$\mathrm{Mn}{\left(\mathrm{OH}\right)}_{3}+e^{-}\to \mathrm{Mn}{\left(\mathrm{OH}\right)}_{2}+{\mathrm{OH}}^{-}\left(E\;\sim -91\;\mathrm{mV}/\mathrm{SCE}\right)$$

According to the time dependence of the spontaneous corrosion potential, it is clear that the composition of corrosion products for Mn^(2)^ and Mn^(3)^ substitutions on the surface of the plain hot-dip galvanized steel will be quite different compared to the reference and Mn^(1)^ substitutions. It can be expected that there will be a significant decrease in the amount of the corrosion product CHZ (calcium hydroxyzincate—Ca[Zn(OH)_3_]_2_·2H2O) and other zinc-based corrosion products (ZnO and Zn(OH)_2_). It is clear that the bond strength of reinforcement with concrete can be influenced not only by the presence of specific types of corrosion products but also by their morphology. In the case where hydrogen evolution has been prevented due to the presence of a sufficient amount of MnO_4_^−^, the presence of specific corrosion products can significantly affect the bond strength of hot-dip galvanized reinforcement with concrete [20,42,52,55,56].

### 3.3. Modified Bond Strength Test

**Figure 9:**
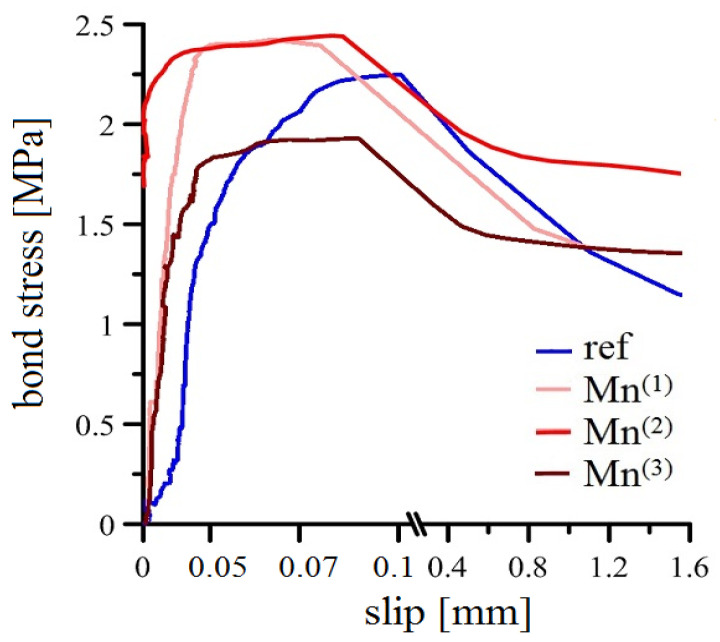
Results of modified bond strength tests (overview; curves—mean values).

**Figure 10:**
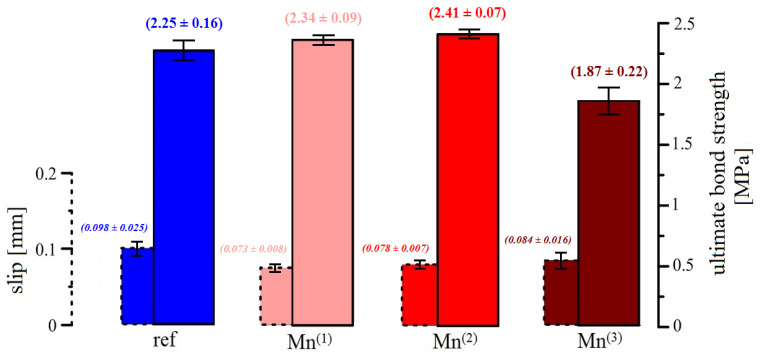
Summary bar chart of bond strength and load-displacement results.

The results of the modified pull-out test are summarized in Figure 9 and Figure 10. Figure 9 shows the average bond stress versus slip curves for a set of specimens embedded in cement paste without MnO_4_^−^ (ref) and with a graded addition of this oxidant (Mn^(1)^–Mn^(3)^). Subsequently, the bar chart in Figure 10 evaluates the measured average ultimate bond strength values (solid line bar boundary; larger bar width) as well as the measured average slip readings for the ultimate bond strength determination (dashed line bar boundary; smaller bar width).

The results of the ultimate bond strength for the reference samples (without KMnO_4_) show a large variability in the data with an average value of 2.25 MPa. While this shear stress was read at the highest slip values (average value is around 0.1 mm) with respect to other groups of samples, it is evident that the hot-dip galvanized reinforcement shifts significantly during the load test in the cementitious mixture and the ultimate bond strength is reached at already relatively high slip values. The bond strength of the hot-dip galvanized plain bar with cement is clearly negatively affected by the hydrogen evolution during the initial corrosion reaction (see Equation (2)). As the released hydrogen increases the porosity of the cement paste at the phase interface [14,20,21,22], the adhesion factor f_ad_ (evaluating the level of adhesion of the cement paste to the surface of the steel reinforcement) decreases according to the equation describing the influence of the individual factors on the overall bond force T_c,i_—see Equation (14) [42]. A description of the individual variables from Equation (14) is summarized in Table 7 [42]. Testing of plain hot-dip galvanized steel allows to assess the effect of coating corrosion under hydrogen evolution in fresh concrete and the formation of CHZ crystals on the bond strength of reinforcement with concrete [20,42]. Because the contribution of the mechanical force of mechanical resistance of specific concrete cover layer f_σ_ (see Equation (15)) to the overall bond force (T_c,i_) is very significant, it exceeds not only the adhesion factor (f_ad_) and the friction factor (f_f_) separately, but clearly their joint influence [20,42]. The reason for this is that, in the case of bond strength testing of ribbed reinforcement, the results are significantly influenced by the mechanical properties of the concrete (specific strength class of the concrete) [65,66].

(14) $$T_{c,i}\approx {\;i}_{f_{\mathrm{ad}}}^{A^{b},A^{r}}+i_{f_{f}}^{A^{b}}+i_{f_{\sigma }}^{A^{r}}$$

(15) $$i_{f_{\sigma }}^{A^{r}}\gg i_{f_{\mathrm{ad}}}^{A^{b},A^{r}}+{\;i}_{f_{f}}^{A^{b}}$$

The presence of zinc corrosion products in a thin layer with a majority CHZ (calcium hydroxyzincate) based composition can increase the adhesion factor of f_ad_ [55,67] (crystal growth perpendicular to the reinforcement surface) but on the other hand, it has been experimentally shown that thicker layers of CHZ corrosion products can significantly reduce the bond strength of hot-dip galvanized reinforcement with concrete [20]. On the basis of the observed highest slip value and the relatively low slope of the curve (mean values) of the bond stress versus slip (Figure 9), it is possible to discuss the negative effect of thicker CHZ layers on the self-adhesion (gradual pulling out of the reinforcement from the reference mix due to the action of bond stress).

The addition of KMnO_4_ to the mix (in the case of Mn^(1)^ and Mn^(2)^ mixes) clearly reduces the slip values for achieving higher ultimate bond strength values. However, from a statistical point of view (see Figure 10), it is clear that only the addition of MnO_4_^−^ anions at a concentration of 10^−3^ mol·L^−1^ ensures already unambiguously higher values of ultimate bond strength (found at lower slip values) than that in the case of the reference exposure. The findings correlate well with the recently published results on the complete blocking of RDHE in hot-dip galvanized steel due to the presence of KMnO_4_ at a concentration of 10^−3^ mol·L^−1^ in simulated concrete pore solutions [37,52]. The observed higher ultimate bond strength values (read for lower slip values) for the Mn^(2)^ substitution compared to the reference exposure are related not only to the suppression of RDHE but also to the formation of less dense and, at the same time, less crystalline corrosion products (corrosion products rich in phases with Mn^II^/Mn^III^ [52]). However, based on the tests performed, it is not possible to decide which of these factors (complete suppression of RDHE, completely different composition of corrosion products compared to the reference exposure) has a more significant effect on the bond strength of hot-dip galvanized steel with concrete. With respect to Equation (14), the effect of surface roughness (f_f_) of hot-dip galvanized plain steel should also be discussed. It is clear from the literature that hot-dip galvanizing very often increases the surface roughness of steel [68,69]. However, it cannot be ruled out that changing the composition of corrosion products can significantly reduce the surface roughness of hot-dip galvanized plain bars. In view of this fact, the adhesion factor f_ad_ may have a much more significant effect on the overall bond strength of hot-dip galvanized plain bars with cement paste than the factor accounting for surface roughness (f_f_)—see Equation (16) [42].

(16) $$i_{f_{\mathrm{ad}}}^{A^{b},A^{r}}\gg i_{f_{f}}^{A^{b}}$$

In the case of addition of KMnO_4_ at a concentration of 10^−2^ mol·L^−1^ (Mn^(3)^ substitution), there is a significant decrease in the ultimate bond strength values and prolongation of slip values. Lower ultimate bond strength values were measured for this mixture than for the reference exposure. Since it is evident that also the addition of 10^−2^ mol·L^−1^ MnO_4_^−^ anions to the simulated concrete pore solution at pH 12.8 completely suppresses RDHE, it is necessary to consider that a significant change in the composition of corrosion products in favor of manganese-rich amorphous phases (mainly Mn^III^ [52]) significantly reduces the adhesion factor (f_ad_). A reduction in the surface roughness (f_f_ effect) of the hot-dip galvanized steel due to the ongoing reactions on its surface may also contribute to the decrease in adhesion (as in the case of Mn^(2)^ substitution). It should also be discussed whether such significant additions of KMnO_4_ to the cement paste reduce its mechanical properties. A significant reduction in the mechanical properties of cement paste may be reflected in a decrease in the f_ad_—adhesion factor. It cannot be ruled out that such significant additions of KMnO_4_ adversely affect the actual hydration of the cement and, consequently, the development of the mechanical properties of the cement paste or the concrete itself.

In the following chapters, it is experimentally verified to what extent the actual hydration process of cement is influenced by graded additions of KMnO_4_ to NSC concrete (measurement of the heat of hydration) and also the effect on the compressive cubic strength. Furthermore, a possible change in the composition of the hydration products of cement paste is discussed using X-ray diffraction analysis.

### 3.4. Measuring of Hydration Heat of Concrete Samples

Hydration of Portland cement is a complex and exothermic process—up to 500 J/g is released during hydration [70]. Since the conductivity of concrete is relatively small, the heat release causes heating of the concrete test bodies. The concept of measuring hydration heats within a semi-adiabatic boundary conditions arrangement (inside an insulated mold) is a conventional technique for verifying the progress of the hydration process [71,72]. Both the quantity of heat as well as the rate at which this heat is evolved depends on the mineralogical and morphological characteristics of cement clinker and depends on a number of factors, mainly water/cement ratio, particle size distribution, specific surface area of the cement, the temperature, and (among others) chemical admixtures [73,74]. The addition of some substances can significantly accelerate the hydration process (e.g., SO_4_^−^) or, on the contrary, hinder it. Important retarders of cement hydration include some sugars [74,75], organic polymers [76,77], or some inorganic substances—ZnO [78,79,80], PbO [80,81], or, e.g., SnCl_2_ [82]. In the case of the latter inorganic substances, the ability of these substances to coat the grains of the hydrated phases (gelatinous surface layer) and, thus, significantly slow down the process is discussed in the older literature in the context of retardation of cement setting and hardening [78,79]. Currently, it is rather accepted that the formation of calcium hydroxyzincate—CHZ (or calcium hydroxyplumbate or calcium hydroxystannate) consumes Ca^2+^ and (OH)^−^ ions from solution and delays the supersaturation and precipitation of calcium hydroxide and development of CSH gel [82].

By measuring the time dependence of the development of the hydration heat, it can be verified that the addition of KMnO_4_ to the mixture does not affect the hydration of the cement. From the measured data, it is possible to discuss the degree of decrease (though an increase is also possible) in the maximum value of the hydration heat released, and from the time course of the curve, it is possible to discuss the time course of the hydration process.

When fresh concrete containing KMnO_4_ was mixed in mortar water, a slight change in consistency was observed compared to the reference mortar without the addition of this substance. The consistency of the fresh concrete was determined according to EN 12350-2, Part 2: Settling test [83]. The cone settlement value of the reference mix was 40 mm, corresponding to a consistency level of S1 according to EN 206 + A2 [84]. The cone settlement values of the material containing KMnO_4_ were 50 mm (Mn^(1)^ and Mn^(2)^) to 60 mm (Mn^(3)^), corresponding to a consistency level of S2. The addition of KMnO_4_ to the mortar water may have a slight plasticizing effect but is more likely to result in so-called concrete bleeding [85,86]. However, there is no significant dewatering, even in Mn^(3)^ mixtures, which would result in a negative effect on the mechanical and physical parameters of the resulting hardened concrete.

**Figure 11:**
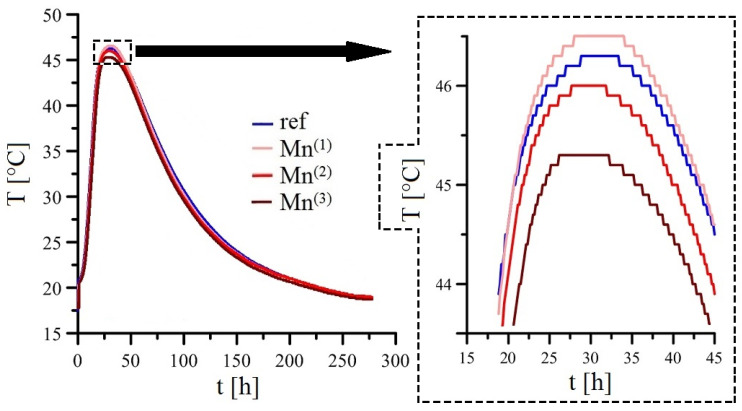
Temperatures of hydrating concrete samples without addition or with addition of KMnO_4_ (overview; curves—mean values).

Figure 11 shows the results of the measurement of the temperature change of the concrete samples during hydration. The results shown are the average curves obtained from parallel groups of test specimens from concrete mixtures without MnO_4_^−^ and with graded additions of these anions. From the measured results, it is clear that additions of KMnO_4_ to the premixed water in the range of 10^−4^ mol·L^−1^ and 10^−3^ mol·L^−1^ have no effect on the evolution of the heat of hydration during cement hydration (with respect to the reference exposure). This is evident both in terms of the measured maximum temperature values reached by the concrete samples and the coincident time at which these global maxima are measured, as well as in terms of the progression of the curve at later time points during the hydration of the concrete samples. In the case of the highest addition of MnO_4_^−^ anions to the mortar water (10^−2^ mol·L^−1^—Mn^(3)^), there is a slight decrease in the maximum temperatures reached by the concrete samples compared to the other mortars by approximately 6%. However, the time course of the temperature evolution curve for the test samples of the mortar with the highest content of KMnO_4_ in the mortar water indicates the same hydration time and the actual hydration process as for the other mortars. It is clear that the highest additions of KMnO_4_ to concrete samples slightly affect the acceleration period (accelerated heat evolution before maximum (first) peak); the effect on the dormant period of the heat evolution is already quite negligible [87].

The evaluation of the cement hydration process (hydration heat) with respect to the time dependence of the temperature evolution (or the evolution of the hydration heat) is a conventional method of monitoring the hydration process. The global maxima of the curve as well as the actual hydration heat evolution curve, are evaluated [88,89].

It is clear that the actual hydration process of Portland cement is only slightly affected by the highest additions of KMnO_4_ to the premixed water (10^−2^ mol·L^−1^) used in this study. However, based on the observed results, it cannot be assumed that MnO_4_^−^ anions, or their reduced forms (MnO_2_, MnO(OH), Mn_2_O_3_, Mn(OH)_2_, Mn_3_O_4_) are able to consume Ca^2+^ and OH^−^ ions to form insoluble Ca_x_[Mny(OH)_z_]_a_·b(H_2_O) complexes and, thus, retard the hydration process. It can also be ruled out that the phases formed were capable of forming gel layers around the clinker phase grains preventing the grains from hydrating themselves. These facts would be more significantly reflected in the evolution of the hydration heat.

Compared to, for example, chloride anions (which significantly promote the hydration process), MnO_4_^−^ additions seem to affect the hydration of tricalcium silicate (C_3_S) and tricalcium aluminate (C_3_A) only marginally [90].

The described decrease in the maximum measured temperature (maximum measured heat) of concrete samples with the addition of 10^−2^ mol·L^−1^ KMnO_4_ is more likely due to the already significant bleeding effect of concrete, which will be more significant, especially in the center of the test samples and in areas with locally increased concentration of dissolved MnO_4_^−^ anions. It is clear that different real w/c ratios affect the hydration process [91]. On the other hand, it cannot be completely ruled out that a slight bleeding effect of concrete occurs due to the MnO_4_^−^ reduction reactions described above.

### 3.5. X-ray Diffraction Analysis of Concrete Samples

Other cement setting retardants also include (besides the above-mentioned) organic substances based on humic acids [92], polyphenolic tannins [93], and starch [94]. Some of these compounds have been shown to form a monomolecular layer on the surface of cement grains and block the hydration process. It is usually considered that the blocking of the hydration process takes place on C_3_A grains [94].

**Figure 12:**
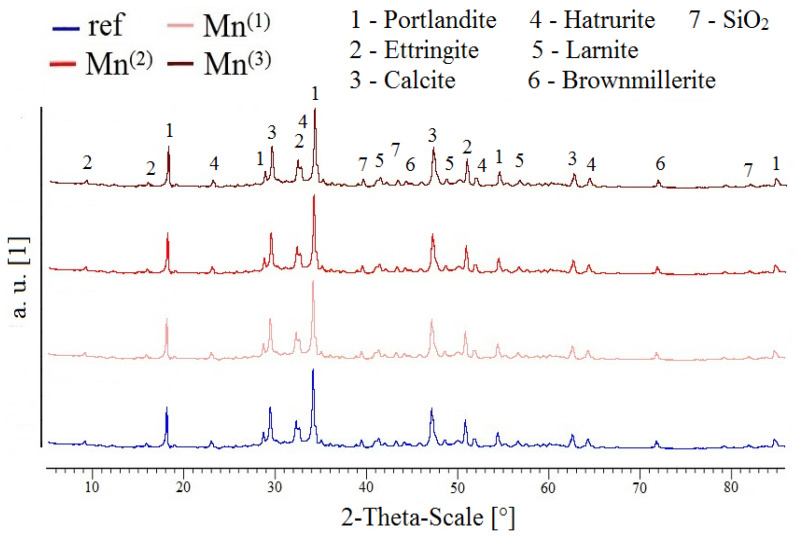
XRD patterns of cement paste samples with different levels of MnO_4_^−^ addition (28 days of curing).

To verify possible changes in the composition of the hydrated phases of the cement paste in the case of KMnO_4_ additions, a comparative XRD analysis was performed on the paste samples after 28 days of maturation. The results of the actual X-ray diffraction analysis are summarized in Figure 12. It is clear from the results that there is no obvious change in the composition of the cement paste after this exposure, even with the addition of 10^−2^ mol·L^−1^ (Mn^(3)^) to the premixed water. In situ X-ray diffraction has been successfully used to evaluate the retardation effect of polymeric substances on cement hydration [77].

In all likelihood, the added MnO_4_^−^ anions can only be consumed during the actual corrosion process of the hot-dip galvanized steel in the concrete (RDHE retardation) and not during the hydration of the cement. Respectively, it can be concluded that even the highest additions of this substance to the cement paste reported in this study do not significantly affect the hydration of the cement. No new phases are formed, nor can it be discussed that there is a significant reduction in the formation of conventional hydration phases.

It is very likely that the MnO_4_^−^ anions present will gradually leach out of the cementitious sealant (concrete), and with regard to the hydration of the cement (concrete) and its subsequent hardening and curing, the inactive KMnO_4_ will be leached out by any flowing water. It cannot be ruled out that less soluble reducing forms of Mn^VII^, such as MnO_2_, MnO(OH), etc., will accumulate as inactive particles in the cement paste (concrete).

### 3.6. Compressive Strength Test of Concrete Cubic Samples

**Figure 13:**
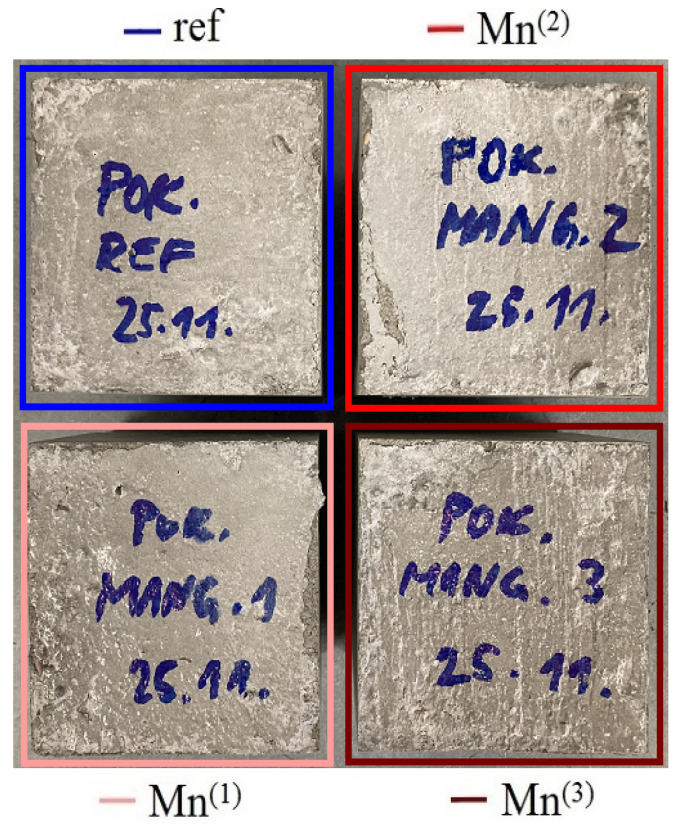
Appearance of homogeneous cube groups from concrete mixtures without (ref) and with MnO_4_^−^ additions for compressive strength test.

To verify the effect of the addition of MnO_4_^−^ on the mechanical properties of normal strength concrete (NSC), compressive strength tests were also carried out on a group of parallel cube specimens after the addition of graded amounts of KMnO_4_ to the mortar water (see Figure 13). Again, it is evident that the addition of KMnO_4_ to the concrete does not change the surface color of the resulting specimens.

**Figure 14:**
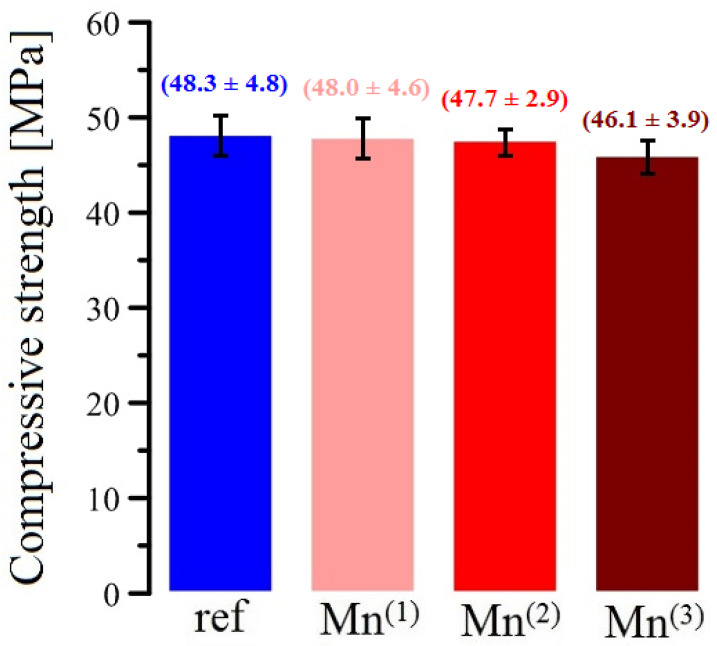
Comparison of compressive cubic strength of NSC samples with different levels of MnO_4_^−^ addition.

The results of this test are shown in the bar graph in Figure 14. It is clear from the results that the addition of MnO_4_^−^ anions in the range of Mn^(1)^ and Mn^(2)^ substitutions does not significantly affect the compressive strength of the resulting samples. At an addition of 10^−2^ mol·L^−1^ KMnO_4_ to the water (Mn^(3)^), a statistically slight decrease in the compressive strength of the resulting concrete is already observed. It is very likely that the slight dewatering in the case of Mn^(3)^ admixture may already affect the w/c value, especially at the surface of the concrete cubes, which may result in a slight decrease in the compressive strength of the concrete bodies, thus formed [95,96]. The significant oxidation potentials of MnO_4_^−^ and the formation of the very stable MnO_2_ phase described above cannot be expected to affect the hydration process of silicate cement with an impact on the compressive strength of the concrete samples. To date, it has not been satisfactorily explained how conventional silicate cement hydrates in the presence of KMnO_4_ additions. It is essential that further research focuses on this topic.

## 4. Conclusions

The results of this paper directly in real cement mixtures confirm the conclusions on the retardation of RDHE (rapid dissolution and hydrogen evolution) for hot-dip galvanized steel in the case of the addition of graded amounts of KMnO_4_ to the mortar water. The effective concentration of the added MnO_4_^−^ anions to the premixed water was experimentally determined to be 10^−3^ mol·L^−1^, since it was found that no corrosion under hydrogen evolution of the hot-dip galvanized steel occurs during the entire exposure time in fresh cement paste. It was further confirmed for this addition of KMnO_4_ that the group of samples of plain hot-dip galvanized steel showed the highest values of ultimate bond strength and the lowest values of slip in the cement paste compared to the reference group (without MnO_4_^−^). In this case, the formation of specific corrosion products on the surface of the hot-dip galvanized steel can also have a positive effect on bond strength.

Additions of MnO_4_^−^ anions to concrete in the range of 10^−4^ mol·L^−1^ are insufficient in terms of limiting the corrosion of HDG specimens under hydrogen evolution and ensuring an undiminished bond strength value compared to the reference group.

Conversely, additions of KMnO_4_ in the range of 10^−2^ mol·L^−1^ cause a decrease in the bond strength of hot-dip galvanized plain bars steel with cement paste. Under such high addition of potassium permanganate, the composition of corrosion products on the surface of the hot-dip galvanized steel is likely to change in favor of Mn^II^/Mn^III^-rich phases, which negatively affect the overall bond strength with cement paste.

By measuring the time evolution of the hydration heat of the concrete samples and also by measuring the compressive strength of the concrete samples, it was verified that the addition of MnO_4_^−^ in the range of 10^−2^ mol·L^−1^ already had a slightly negative effect on the hydration of the cement and also on the evolution of the compressive strength of the resulting concrete. These additions already lead to a slight water separation, which results in a slight bleeding of the concrete. This can also have a negative effect on the bond strength. 

It is very likely that the added KMnO_4_ is partially consumed during the actual corrosion process (RDHE limitation) without significant changes in the composition of the hydrated phases of the cement. 

The use of KMnO_4_ as a concrete additive to increase the efficiency of the use of hot-dip galvanized concrete reinforcement (RDHE limitation) is apparently complicated by the need to ensure a sufficient concentration of this additive and, at the same time, to avoid excessive dosage (affecting the mechanical properties of the concrete and its own cohesion). At the same time, it cannot be ruled out that the release of manganese oxo-hydroxides from the concrete by leaching may occur.

## Figures and Tables

**Table 1:** Cement composition guaranteed by producer (CEM I 42.5 R).

Compound	CaO	SiO_2_	Al_2_O_3_	Fe_2_O_3_	MgO	SO_3_	K_2_O	Na_2_O
content (wt. %)	64.2	19.5	4.7	3.2	1.3	3.2	0.78	0.09

**Table 2:** Content by m^3^ of concrete for X-ray diffraction analysis and measuring of hydration heat.

Mixture	Portland Cement (kg)	Water (L)	Gravel (kg)	Sand (kg)
w/c: 0.35	14	4.9	0	0

**Table 3:** Content by m^3^ of concrete cubic samples for compressive strength measuring.

Admixture	Content (kg/m^3^)	Note
cement (CEM I 42.5 R)	365	pure Portland cement
aggregate	900	fraction 0/4—fine sand
	585	fraction 4/8
	285	fraction 8/16
mixture (w/c)	0.55	

**Table 4:** Detailed characterization of the amount of KMnO_4_ addition to the mixtures of all concrete samples.

Marking of Mixtures in This Article	Used Cement	Addition of KMnO_4_ to the Mixing Water
ref	CEM I—42.5 R (Portland cement—pure)	without addition
Mn^(1)^	CEM I—42.5 R (Portland cement—pure)	with addition 10^−4^ mol·L^−1^
Mn^(2)^	CEM I—42.5 R (Portland cement—pure)	with addition 10^−3^ mol·L^−1^
Mn^(3)^	CEM I—42.5 R (Portland cement—pure)	with addition 10^−2^ mol·L^−1^

**Table 5:** Comparison of potentials between a plate of stainless steel (FeCr18Ni9) and SCE for simulated pore solutions of concrete with graded amounts of MnO_4_^−^ anions.

Simulated Concrete Pore Solution	Composition of Simulated Concrete Pore Solution	Difference in Potencial Values (FeCr18Ni9)/SCE	Average Value for the E_corr_/SCE Expression
ref	pH 12.8	20–50 mV	35 mV
Mn^(1)^	pH 12.8 + 10^−4^ mol·L^−1^ MnO_4_^−^	30–50 mV	40 mV
Mn^(2)^	pH 12.8 + 10^−3^ mol·L^−1^ MnO_4_^−^	75–105 mV	90 mV
Mn^(3)^	pH 12.8 + 10^−2^ mol·L^−1^ MnO_4_^−^	125–155 mV	140 mV

**Table 6:** Composition of plain bar steel 10216 (GD-OES).

Compound	C	Al	Si	P	S	Cr	Mn	Cu	Zn	Fe
content, (wt. %)	0.11	0.02	0.23	0.01	0.02	0.06	0.55	0.22	0.01	balance

**Table 7:** Description of individual variables from Equation (14).

Symbol	Property	Unit
T_c,i_	bond force	N
i	contribution of force	N
f_ad_	completed adhesion force	N
f_f_	friction force	N
f_σ_	force of mechanical resistance of specific concrete cover layer	N
A^b^	total area of bar body	m^2^
A^r^	total area of bar ribs	m^2^

## Data Availability

Data is contained within the article.
